# Sarcopenia and Menopause: The Role of Estradiol

**DOI:** 10.3389/fendo.2021.682012

**Published:** 2021-05-19

**Authors:** Annalisa Geraci, Riccardo Calvani, Evelyn Ferri, Emanuele Marzetti, Beatrice Arosio, Matteo Cesari

**Affiliations:** ^1^Geriatric Unit, Fondazione IRCCS Ca’ Granda Ospedale Maggiore Policlinico, Milan, Italy; ^2^Fondazione Policlinico Universitario “Agostino Gemelli” IRCCS, Rome, Italy; ^3^Aging Research Center, Department of Neurobiology, Care Sciences and Society, Karolinska Institutet and Stockholm University, Stockholm, Sweden; ^4^Institute of Internal Medicine and Geriatrics, Università Cattolica del Sacro Cuore, Rome, Italy; ^5^Department of Clinical Sciences and Community Health, University of Milan, Milan, Italy; ^6^Geriatric Unit, IRCCS Istituti Clinici Scientifici Maugeri, Milan, Italy

**Keywords:** aging, endocrinology, hormones, menopause, muscle

## Abstract

During aging and menopausal transition in women, a progressive muscle degeneration (*i.e.* decrease in quality and muscle function) occurs. This muscle dysfunction, caused by decreased proliferation of muscle satellite cells, increased levels of inflammatory markers, and altered levels of sex hormones, exposes women to a raised incidence of sarcopenia. In this regard, hormonal balance and, in particular, estradiol, seems to be essential in skeletal muscle function. The role of the estradiol on satellite cells and the release of inflammatory cytokines in menopausal women are reviewed. In particular, estradiol has a beneficial effect on the skeletal muscle by stimulating satellite cell proliferation. Skeletal muscle can respond to estrogenic hormonal control due to the presence of specific receptors for estradiol at the level of muscle fibers. Additionally, estradiol can limit inflammatory stress damage on skeletal muscle. In this review, we primarily focused on the role of estradiol in sarcopenia and on the possibility of using Estradiol Replacement Therapy, which combined with nutritional and physical activity programs, can counteract this condition representing a valid tool to treat sarcopenia in women.

## Introduction

Sarcopenia is a typical condition of the aging processes that is characterized by decline in muscle mass and quality (1). The sarcopenia onset is determined by hormonal changes, activation of the inflammatory pathway, fat infiltration, apoptosis, and altered mitochondrial function (2). The incidence of some common geriatric syndromes is sex-specific and, in particular, the onset of sarcopenia in women seems to be intimately linked to menopause (3).

One of the most striking phenomena marking women’s aging process is menopause, which brings about hormonal changes (4, 5) and, in particular, estradiol levels. Estradiol is the most potent estrogen hormone. It regulates the menstrual cycle and is responsible for the development and maintenance of female sexual characteristics. Interestingly, the skeletal muscle possesses specific estradiol receptors at the fiber levels. Therefore estradiol can promote muscle regeneration stimulating the proliferative activity of muscle satellite cells and contributing to muscle health (6, 7).

Muscle satellite cells represent the skeletal muscle stem cells that are responsible for muscle tissue maintenance. Following mechanical stress (*e.g.*, physical exercise) or muscle damage, these cells activate their regenerative function, rebuilding integrity and muscle function (8).

**Figure 1:**
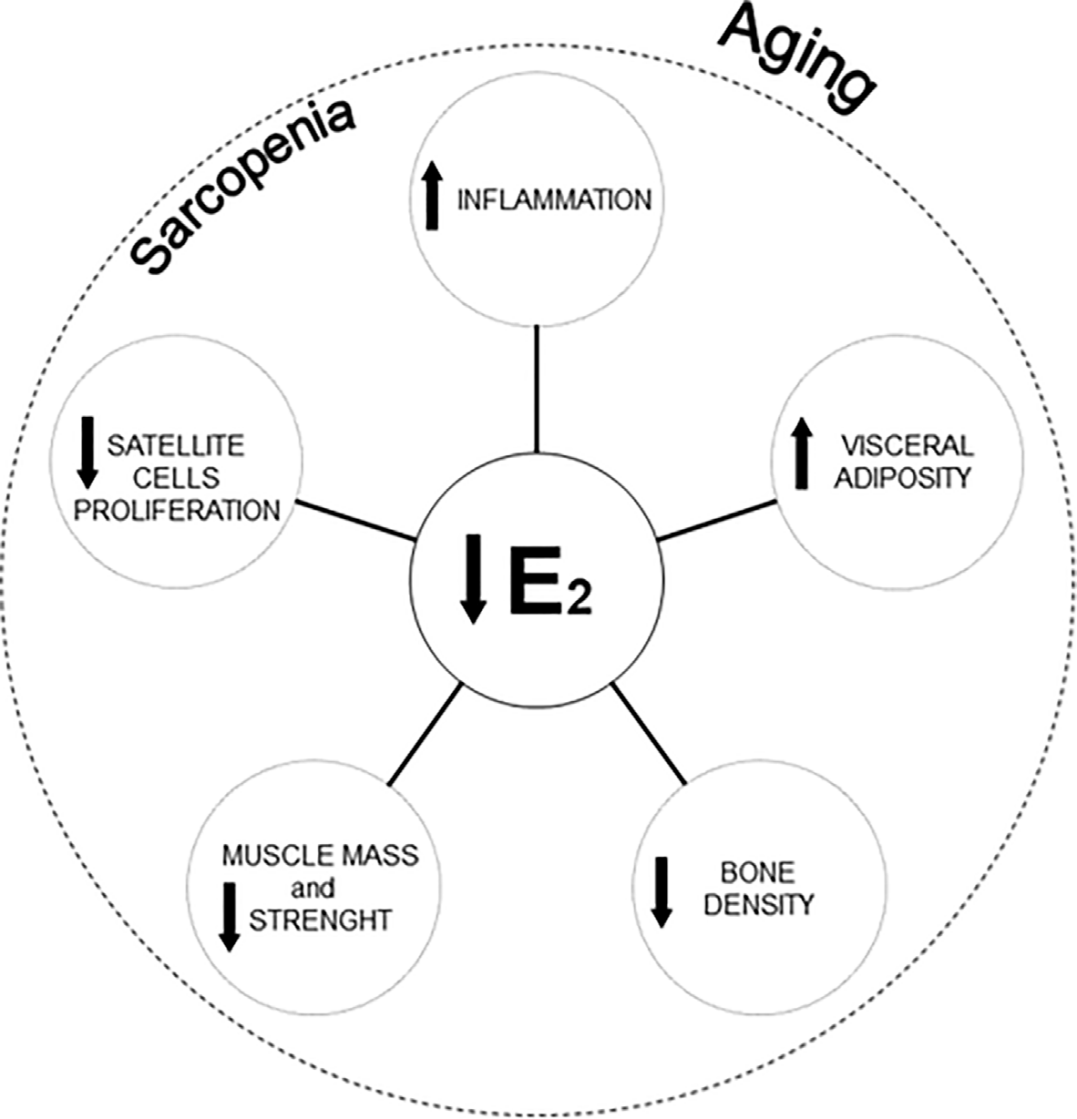
Reduction of estradiol concentrations, morphological changes, and cellular modifications affecting sarcopenia in women aging. E2, Estradiol.

It is to note that the menopausal transition (staged as pre-menopause, perimenopause, menopause, and post-menopause) (9) is associated not only with a decline in estradiol levels (10), but also with an increased visceral adiposity and decreased bone density, muscle mass, and muscle strength (7) (**Figure 1**). All these factors significantly contribute to the development of a condition termed “sarcopenic obesity” (11) characterized by a sarcopenic clinical condition and an excessive body weight. Sarcopenic obesity has direct consequences on the health of menopausal and post-menopausal women (12).

Interestingly, estradiol is also involved in the modulation of the local and systemic inflammatory responses thus affecting sarcopenia (13, 14).

In this review, we will discuss the role of serum estradiol concentrations on the incidence of sarcopenia in menopausal women. In particular, we will provide a brief overview of the estradiol effects on muscle satellite cells and the release of inflammatory cytokines, hypothesizing a possible target for sarcopenia interventions in women.

## Estradiol Deficit and Inflammation

Evidence suggests that menopause is closely associated with an increased release of pro-inflammatory cytokines, such as interleukin (IL)-6, IL-1, and tumor necrosis factor-α (TNF-α) (15).

Notably, some studies have suggested how adipocytes, through the release of IL-6 and TNF-α, can promote the accumulation of fat mass and compromise muscle function (16–18), thus causing sarcopenia (19–21).

Although there is a clear correlation between increased peripheral IL-6 and TNF-α levels with sarcopenia, the causes and mechanisms remain partially unclear.

Nonetheless, studies have shown the capability of 17*β*-estradiol to specifically inhibit the inflammation-mediated release of some pro-inflammatory cytokines, like TNF-α (22), which can degrade muscle proteins and reduce the ability of the adult muscle to respond to damage (23).

An exciting aspect resides in the ability of estrogen treatment to attenuate fat gain and decreased lean mass, modulating the inflammation in the skeletal muscle and thus reducing the risk of “sarcopenic obesity” (12, 24). In this regard, there are convincing lines of evidence on the ability of estrogens to act and influence adipose tissue directly through the estrogen receptor-*α* (16).

Under these premises, the fact that estrogen can inhibit the inflammatory response could be of extreme interest to prevent further damage (25).

## Role of Estradiol in Skeletal Muscle Stem Cell Physiology

Muscle fibers house a population of stem cells (satellite cells), ensuring plasticity and regeneration (26). These cells are abundant during the early phases of development, contributing to muscle growth and then decrease over time (27). Their content varies depending on fiber types. Type I oxidative fibers have a higher content of satellite cells than type II fibers because they benefit from more significant blood and capillary contribution (26).

In steady-state conditions, satellite cells are in a quiescent state (28, 29). Following muscle injury or anabolic stimulation, they are activated and enter the myogenic program to support the repair of muscle damage through fiber repair or growth (30, 31)

The age-related chronic inflammatory state impacts the proliferation and the replenishment of satellite cells (26). In this regard, the role played by estradiol becomes of considerable interest. Estradiol stimulates the activation and, consequently, the proliferation of satellite cells through specific estrogen receptors (ER; *e.g.*, ER-*α* and ER-*β*) (32) (**Figure 1**), promoting muscle repair (5, 7, 13, 33).

Nevertheless, the ability of estradiol to make the skeletal muscle generating force (13) seems to exclusively depend on the binding of this hormone with the receptor-*α* (13, 34).

## Future Perspectives

There is convincing evidence that estrogens and, especially, estradiol play a key role in the preservation of muscle health in old age (35).

In this regard, several research studies on interventions aimed at hormone replacement have been conducted. In some cases, hormone supplementation (or HRT, Hormone Replacement Therapy) with estradiol has generated enormous interest for its potentially beneficial effects (36, 37). Specifically, menopause-related obesity and loss of lean and skeletal muscle mass have been shown to reverse following estradiol hormone therapy (38).

In addition, estrogen replacement has shown different effects by performing it in a specific phase of menopause. Specifically, the use of HRT in the “initial post-menopause” compared to a “delayed post-menopause” phase resulted in a significant increase in the number of muscle satellite cells (39) as well as in an improvement of mobility and muscle strength (40).

Conversely, it should be noted that some studies have considered Estradiol Replacement Therapy (ERT) an ineffective method against muscle loss (35). Others have found an association of hormone therapy with an increased risk of breast cancer (41) and/or cardiovascular disease (42).

Nonetheless, the effects of hormone therapy still remain, however, controversial. For all these reasons, lifestyle interventions (in particular physical activity and nutritional interventions) currently remain the cornerstones for maintaining muscle health into advanced age (43). Large research programs [*e.g.*, the Sarcopenia and Physical fRailty IN older people: multi-componenT Treatment strategies (SPRINTT) project] (44, 45) have been conducted to define the critical steps for facilitating the development of pharmacological interventions against sarcopenia. Researchers are defining the regulatory framework for offering (pharmacological) opportunities to treat or prevent sarcopenia and its adverse outcomes (46).

Women not adequately responding to lifestyle intervention, for example, could combine a physical-rehabilitation and nutritional program with ERT to improve their health condition for preserving muscle mass and function into late life. Of course, studies supporting this hypothesis are needed.

## Discussion

Although the mechanisms related to muscle loss in menopausal and post-menopausal women require further studies, the idea of using ERT in addition to nutritional and physical programs for tackling sarcopenia might be interesting to explore. The fact that inflammation is positively influenced by estradiol may provide a solid biological background to the hypothesis. Novel therapeutic perspectives based on the estradiol effects might become fundamental in the future to prevent/delay the development of sarcopenia in aging women.

## Author Contributions

AG, EM, and MC drafted the manuscript. RC, EF, and BA provided critical revision. All authors contributed to the article and approved the submitted version.

## Conflict of Interest

The authors declare that the research was conducted in the absence of any commercial or financial relationships that could be construed as a potential conflict of interest.

The reviewer BS declared a shared affiliation with some of the authors, RC and EM, to the handling editor at time of review.
